# Combining Viral Vectored and Protein-in-adjuvant Vaccines Against the Blood-stage Malaria Antigen AMA1: Report on a Phase 1a Clinical Trial

**DOI:** 10.1038/mt.2014.157

**Published:** 2014-09-30

**Authors:** Susanne H Hodgson, Prateek Choudhary, Sean C Elias, Kathryn H Milne, Thomas W Rampling, Sumi Biswas, Ian D Poulton, Kazutoyo Miura, Alexander D Douglas, Daniel GW Alanine, Joseph J Illingworth, Simone C de Cassan, Daming Zhu, Alfredo Nicosia, Carole A Long, Sarah Moyle, Eleanor Berrie, Alison M Lawrie, Yimin Wu, Ruth D Ellis, Adrian V S Hill, Simon J Draper

**Affiliations:** 1 The Jenner Institute Laboratories, University of Oxford, Oxford, UK; 2 Centre for Clinical Vaccinology and Tropical Medicine, The Jenner Institute, University of Oxford, Churchill Hospital, Oxford, UK; 3 Laboratory of Malaria and Vector Research, NIAID/NIH, Rockville, Maryland, USA; 4 Okairòs, Rome, Italy; 5 CEINGE, Naples, Italy; 6 Department of Molecular Medicine and Medical Biotechnology, University of Naples Federico II, Naples, Italy; 7 Clinical Biomanufacturing Facility, University of Oxford, Churchill Hospital, Oxford, UK; 8 Laboratory of Malaria Immunology and Vaccinology, NIAID/NIH, Rockville, Maryland, USA

## Abstract

The development of effective vaccines against difficult disease targets will require the identification of new subunit vaccination strategies that can induce and maintain effective immune responses in humans. Here we report on a phase 1a clinical trial using the AMA1 antigen from the blood-stage *Plasmodium falciparum* malaria parasite delivered either as recombinant protein formulated with Alhydrogel adjuvant with and without CPG 7909, or using recombinant vectored vaccines—chimpanzee adenovirus ChAd63 and the orthopoxvirus MVA. A variety of promising “mixed-modality” regimens were tested. All volunteers were primed with ChAd63, and then subsequently boosted with MVA and/or protein-in-adjuvant using either an 8- or 16-week prime-boost interval. We report on the safety of these regimens, as well as the T cell, B cell, and serum antibody responses. Notably, IgG antibody responses primed by ChAd63 were comparably boosted by AMA1 protein vaccine, irrespective of whether CPG 7909 was included in the Alhydrogel adjuvant. The ability to improve the potency of a relatively weak aluminium-based adjuvant in humans, by previously priming with an adenoviral vaccine vector encoding the same antigen, thus offers a novel vaccination strategy for difficult or neglected disease targets when access to more potent adjuvants is not possible.

## Introduction

*Plasmodium falciparum*, the primary causative agent of human malaria, remains the most significant parasitic infection worldwide, responsible for over 200 million infections and upwards of 600,000 deaths in 2012.^1,2^ Development of a highly effective vaccine against *P. falciparum* thus remains an urgent global health priority with the potential to reduce both mortality and transmission.^3^ However, in light of disappointing phase 3 efficacy results reported for the leading pre-erythrocytic vaccine RTS,S in the target infant age group,^4^ there remains a strong rationale to pursue development of alternative vaccine strategies, including those targeting the asexual blood-stage and the sexual transmission-stages of the parasite's lifecycle. Like the liver-invasive sporozoites targeted by RTS,S, these subsequent stages of the lifecycle are susceptible to antibodies—either acting within the blood to prevent red blood cell invasion, or within the mosquito to interfere with sexual development.^5,6^ Development of subunit vaccination strategies that can induce and maintain effective cellular and humoral responses in humans will thus be a primary requisite for success, given the apparent need for extremely high antibody concentrations, against leading target antigens, in order to neutralize malaria parasites.^7,8^

Most blood-stage and transmission-blocking malaria vaccine candidates trialed to-date have been recombinant protein-in-adjuvant formulations. In many cases, these have been designed to induce high-titer functional antibodies that exert growth inhibitory activity (GIA) against merozoite antigens involved in the red blood cell invasion process,^6,9^ or which inhibit sexual development of the parasite within the mosquito,^10^ respectively. However, in a number of cases experimental protein vaccine adjuvants have shown unacceptable safety or reactogenicity profiles in clinical trials,^11,12,13^ moreover, to-date, only a small number of adjuvants have been licensed for human use—including aluminium-based salts (aluminium phosphate and aluminium hydroxide); virosomes; the oil-in-water emulsion MF59 (Novartis), and the adjuvant-system platform AS03 and AS04 developed by GlaxoSmithKline.^14,15^ Similarly, the lack of access to many promising adjuvants developed by some companies has had an adverse effect on vaccine development for difficult diseases, such as malaria, in which there is relatively limited commercial interest and very strong immune responses are required for protection.^16^

In recent years, we have evaluated an alternative approach to subunit vaccination aimed at inducing both cellular and humoral immune responses. In this case, a heterologous prime-boost regimen is used with recombinant adeno- and pox-viral vectors.^17,18^ In order to translate this approach into human clinical trials, we utilized a chimpanzee adenovirus serotype 63 (ChAd63) vector to prime immune responses, followed by a boost 8 weeks later with the orthopoxvirus vector-modified vaccinia virus Ankara (MVA).^19,20^ In a series of phase 1a clinical trials, we showed that vectors recombinant for the blood-stage *P. falciparum* antigens merozoite surface protein 1 (MSP1) or apical membrane antigen 1 (AMA1) can induce Th1-isotype-skewed serum IgG antibody responses and strong T cell and memory B cell (mBC) responses^21,22,23^ (Biswas *et al.*, *PLoS One* (in press)). However, these vaccine candidates failed to demonstrate clinical efficacy, with no impact on blood-stage parasite multiplication rates observed in vaccinated UK adults following controlled human malaria infection administered by mosquito bite.^24^ The vectors targeting both blood-stage antigens induced on average 20–50 µg/ml of antigen-specific serum IgG,^21,22^ and it is likely these antibody concentrations were too low to mediate functional GIA *in vivo*.^7^

More recent preclinical studies have focused on combining viral vectored and protein-in-adjuvant vaccines in “mixed-modality” regimens, with the aim of maximizing the induction of both cellular and humoral immune responses.^18^ In most cases, a recombinant adenoviral vaccine is used to prime the immune response, followed by boosting with MVA and/or protein. Studies of these regimens in mice or rhesus macaques with the MSP1 or AMA1 antigens have shown that these mixed-modality approaches can achieve antibody and T cell responses which equal, or in some cases surpass, the best immune responses achieved by either vaccine technology alone.^25,26^ Priming with viral vectors also led to enhanced IgG antibody avidity and Th1-type IgG isotype skew in mice.^25,27^ Preclinical data from the fields of HIV-1^28,29,30,31,32^ and liver-stage malaria vaccine development^33,34^ also show encouraging results from the combination of adenoviral-vectored and protein-based vaccines. While undertaking the above mouse studies, we also reported that priming with a recombinant adenovirus followed by a booster vaccination with protein-in-adjuvant, greatly reduced the hierarchy of humoral immunogenicity that is typically observed when comparing protein vaccine adjuvants. In this case, relatively weak protein adjuvants such as aluminium-based salts, boosted antibody responses comparably to far more immunogenic formulations, in contrast to results observed when the protein-in-adjuvant vaccines were used alone.^27^ This adenovirus-protein regimen also induced a more cytophilic antibody response, dominated by IgG2a, and improved the efficacy of a weakly immunogenic MSP1 protein vaccine delivered in AdjuPhos (aluminium phosphate) in a *P. yoelii* rodent malaria blood-stage challenge model.^27^ In rhesus macaques, an AMA1 vaccine formulated in Alhydrogel boosted IgG responses comparably to the same vaccine in CoVaccineHT adjuvant following ChAd63-MVA priming.^26^ Overall, these data suggested that the differential immunogenicity of protein-in-adjuvant vaccines may be overcome by prior immunization with recombinant adenovirus. They also indicated that a mixed-modality approach may circumvent the need to use more reactogenic adjuvants to achieve maximal antibody responses.

Here we made use of an opportunity to test the mixed-modality concept in a phase 1a clinical trial, given adenoviral, poxviral and protein AMA1 vaccines were available, and the protein could be administered in Alhydrogel with or without a second potent immuno-stimulator adjuvant (CPG 7909). We thus present the safety and immunogenicity data of this vaccine delivery approach in humans.

## Results

### VAC044 study recruitment and vaccinations

**Figure 1:**
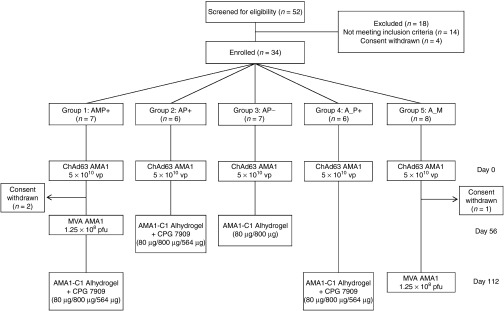
VAC044 flow chart of study design and volunteer recruitment. Eighteen volunteers were excluded following screening for the following reasons: prior malaria exposure (two volunteers); excessive alcohol consumption (one volunteer); proteinuria (one volunteer); positive antinuclear antibody at screening (two volunteers); nickel allergy (two volunteers); anemia (one volunteer); psychiatric history (four volunteers); hematuria (one volunteer); consent withdrawn prior to enrolment (four volunteers). All immunizations were administered intramuscularly with sequential vaccines administered into the deltoid of alternating arms. Two volunteers in Group 1 withdrew from the study 56 days post-ChAd63 AMA1 for personal reasons. One volunteer in Group 5 withdrew from the study 57 days post-ChAd63 AMA1 for personal reasons and was replaced with a new volunteer, thus *n* = 8 recruited into this group. Throughout the paper the immunization regimens are referred to as defined in the Group boxes, *e.g.*, AMP+ = ChAd63 prime, MVA boost, AMA1-C1 protein-in-Alhydrogel + CP7909 boost with 8-week intervals; A_P+ = ChAd63 prime, AMA1-C1 protein-in-Alhydrogel + CP7909 boost with a 16-week interval. Where the “AM” regimen is referred to, this relates to ChAd63 prime, MVA boost with an 8-week interval from Group 1 (before the protein vaccine boost).

The VAC044 study was designed to assess a variety of promising mixed-modality regimens previously tested in mice and rhesus macaques. These included the use of 16 week (as opposed to 8 week) prime-boost regimens,^25,26^ and using protein vaccine formulated in an aluminium-based adjuvant to boost antibody responses primed by an adenoviral vaccine.^27^ Recruitment took place between May 2011 and July 2012. Thirty-four healthy malaria-naive adult volunteers (14 female and 20 male) were enrolled (**Figure 1**). The mean age of volunteers was 26.5 years (range: 19–48). Vaccinations began in June 2011 and all follow-up visits were completed by March 2013. All vaccinees received their immunizations as scheduled with the exception of two volunteers in Group 1 and one volunteer in Group 5 who withdrew from the study for personal reasons following priming with ChAd63 AMA1. Their data were included for the safety analysis, but excluded from immunological analysis. All doses of vaccines were the same as those tested in previous phase 1/2a studies.^22,24,35,36^

### Vaccine safety and reactogenicity

No unexpected or serious adverse events (AEs) occurred and no volunteers were withdrawn due to AEs. ChAd63 AMA1 administered at the dose of 5 × 10^10^ vp (*n* = 34) on day 0 demonstrated a favorable safety profile, similar to that seen in the previous phase 1/2a studies,^22,24^ with the majority of AEs mild in severity (82%), all resolving completely (**Supplementary Figure S1**). One volunteer (3%) experienced severe feverishness, myalgia, and malaise postvaccination which resolved within 72 hours.

MVA AMA1 administered at the dose of 1.25 × 10^8^ pfu on day 56 (Group 1) or day 112 (Group 5) was well tolerated (*n* = 12) with the majority of AEs experienced mild in severity (83%) (**Supplementary Figure S2**). As seen with MVA AMA1 and other MVA vectored vaccines,^21,22,24^ the majority of volunteers experienced injection site pain (92%), which was moderate or severe in intensity in 58% of volunteers. Five volunteers (42%) experienced at least one systemic AE that was moderate or severe in intensity. All resolved within 96 hours with the exception of a moderate exacerbation of previously quiescent childhood asthma for which the volunteer was still receiving treatment at the end of the study. No difference was seen in the local or systemic reactogenicity profile of MVA AMA1 when administered 8 or 16 weeks after ChAd63 AMA1 (*i.e.*, between Groups 1 and 5).

As seen in a previous study of malaria-naive volunteers,^36^ the addition of CPG 7909 to AMA1-C1/Alhydrogel increased reactogenicity, especially systemic AEs, however both vaccines were overall well tolerated (**Supplementary Figure S3**). Priming with ChAd63 AMA1 (with or without MVA AMA1 booster) did not appear to significantly change the reactogenicity of AMA1-C1/Alhydrogel ± CPG 7909, *i.e.*, when comparing between Groups 1 and 2, or when comparing safety data with those from other studies including malaria-naive volunteers.^35,36^ No difference was seen in the local or systemic reactogenicity profile of AMA1-C1/Alhydrogel + CPG 7909 administered 8 or 16 weeks after ChAd63 AMA1 (*i.e.*, comparing between Groups 2 and 4).

### T cell responses and phenotype

**Figure 2:**
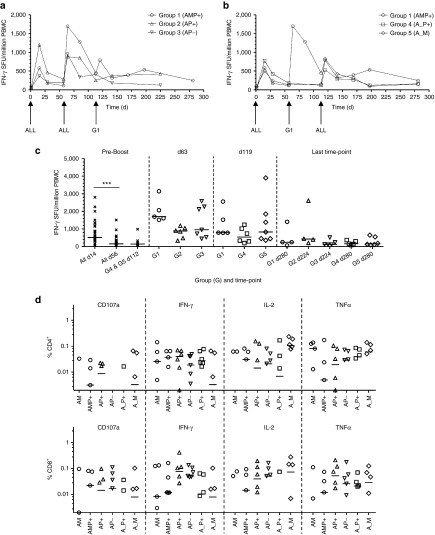
T cell responses of mixed-modality AMA1 immunization regimens. T cell responses were assessed in each group by *ex vivo* IFN-γ ELISPOT using fresh peripheral blood mononuclear cell (PBMC). All volunteers received the same prime with ChAd63 AMA1 on d0. Median responses are shown over time for the 3D7 AMA1 allele in (**a**) Groups 1, 2, and 3 which all received a booster immunization on day 56, and (**b**) Groups 1, 4, and 5 which all received a booster immunization on d112, (note Group 1 received a booster immunization at both of these time-points). (**c**) Median and individual IFN-γ ELISPOT responses are shown for key time-points: all volunteers combined before boosting at days 14 and 56 (*n* = 31) and day 112 (*n* = 13); 1 week after the booster immunizations (d63 and d119); and the final time-point of follow-up 24 weeks after the last immunization (d224 or d280). ***P < 0.0001, Wilcoxon matched-pairs signed rank test. (**d**) Frozen PBMC from 4 weeks post-booster immunization = d84 (AM, AP+, AP−) and d140 (AMP+, A_P+, and A_M), were restimulated with a pool of 3D7 AMA1-specific peptides and assayed by intracellular cytokine staining for all volunteers (except for one in Group 5 (A_M) for which cells were not available). Individual and group median responses are shown for the % CD4^+^ (top) and CD8^+^ (bottom) T cells positive for CD107a, IFN-γ, IL-2, or TNF-α. Any values <0.002% are not shown.

T cell responses provide important help to B cell responses induced by subunit vaccines and in many instances will contribute to protective efficacy against difficult pathogens. T cell responses were thus monitored over time in all groups by *ex vivo* IFN-γ ELISPOT. Median responses in each group are shown (**Figure 2a**,**b**) as well as individual responses at key time-points (**Figure 2c**). All volunteers in the study were primed with the same dose of ChAd63 AMA1, resulting in a median response at day 14 of 518 SFU/million peripheral blood mononuclear cell (PBMC) (range: 74–2,796) (**Figure 2c**), in good agreement with previous studies of the same vaccine.^24^ These responses contracted prior to boosting on days 56 and/or 112. Following boosting on day 56 (d56), significantly increased responses were observed across all three vaccinated groups by d63 when the data from the three groups were combined (*P* = 0.0004, *n* = 18, Wilcoxon matched pairs signed-rank test). MVA AMA1 (Group 1) tended to show the strongest responses post-boost on d63 (median of 1698 SFU/million PBMC) but this did not reach significance (*P* = 0.06, Kruskal–Wallis test), whilst the AMA1-C1/Alhydrogel vaccines boosted comparably irrespective of whether CPG 7909 was included in the formulation (medians of 883 and 952 SFU/million PBMC, respectively) (**Figure 2a**,**c**). Following boosting on d112, significantly increased responses were again observed across all three vaccinated groups by d119 when the data from the three groups were combined (*P* = 0.0002, *n* = 18, Wilcoxon matched pairs signed-rank test). A modest increase was observed in the AMP+ group with a median response of 790 SFU/million PBMC (lower than that observed after the first MVA AMA1 boost at week 8). Responses in the A_P+ and A_M groups showed medians of 538 and 828 SFU/million PBMC respectively, indicating that T cell immunogenicity tended to be lower following a vaccine boost at 16 weeks in comparison to 8 weeks (**Figure 2b**,**c**). Following contraction of all responses into the memory phase, there was no significant difference between any of the regimens, irrespective of number of immunizations, prime-boost interval or adjuvant formulation, by the last time-point 24 weeks postfinal immunization (d224 or d280) (*P* = 0.45, Kruskal–Wallis test) (**Figure 2c**).

The phenotype of the AMA1-specific T cell responses was also assessed by flow cytometry and intracellular cytokine staining for IFN-γ, TNF-α, IL-2, and CD107a (**Figure 2d** and **Supplementary Figure S4**). Frozen PBMC taken 4 weeks after each booster immunization (day 84 for the AM, AP+ and AP− regimens, and day 140 for the AMP+, A_P+, and A_M regimens) were thawed and restimulated with a pool of peptides corresponding to the 3D7 allele of AMA1. In agreement with the *ex vivo* IFN-γ ELISPOT data for these specific time-points (**Figure 2a**,**b**), responses were largely comparable across all the groups. A mixed CD4^+^ and CD8^+^ T cell response was observed following all regimens, suggesting that both the MVA and protein vaccines are capable of boosting these T cell responses primed by ChAd63.

### Antibody responses assessed by ELISA

**Figure 3:**
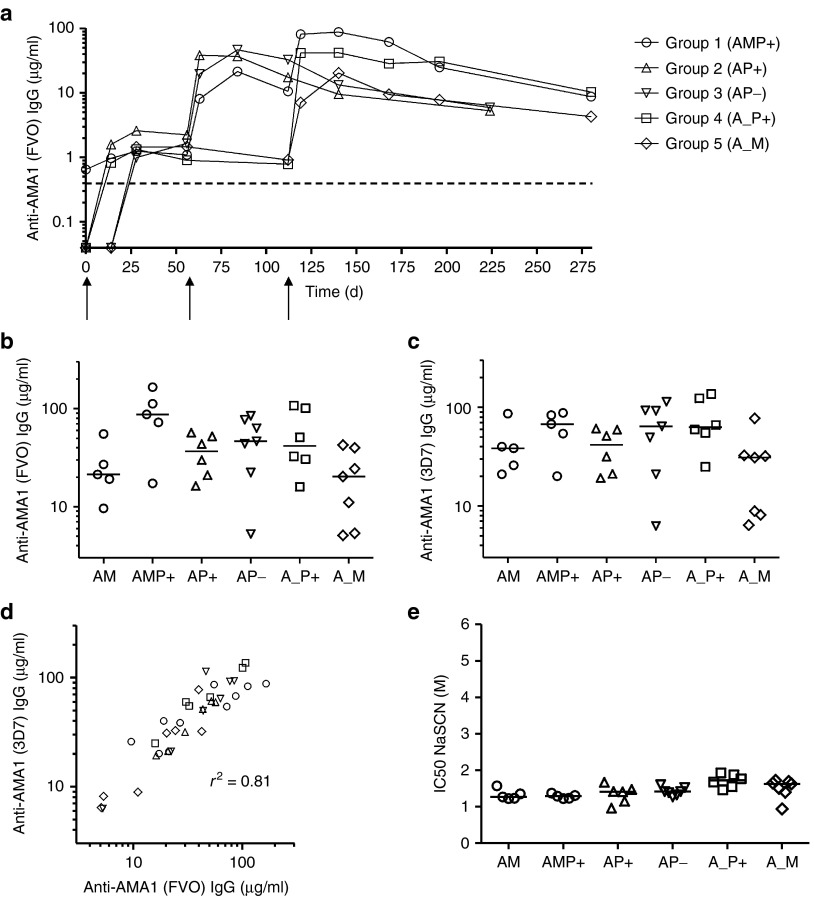
Humoral responses of mixed-modality AMA1 immunization regimens. Serum IgG antibody responses were assessed in each group by anti-AMA1 enzyme-linked immunosorbent assay (ELISA). All volunteers received the same prime with ChAd63 AMA1 on d0. (**a**) Median responses are shown over time for all groups for the FVO AMA1 allele. The dashed line indicates the limit of detection in the assay. (**b**) Median and individual ELISA responses against FVO AMA1 are shown four weeks after all booster vaccinations: day 84 following AM, AP+, and AP− immunization, and day 140 following AMP+, A_P+, and A_M immunization. (**c**) The same as in **b** except the ELISA was performed for 3D7 AMA1. (**d**) Concordance between the anti-AMA1 total IgG ELISA readouts between the two allelic variants of AMA1 (responses as shown in **b** and **c**). Linear regression *r*^2^ value is shown; slope = 0.96 (95% CI: 0.79–1.13); Y-intercept = 8.9 µg/ml (95% CI: 0.23–17.6) (*n* = 36). (**e**) Avidity of serum IgG responses was assessed by NaSCN-displacement 3D7 AMA1 ELISA and is reported as the molar (mol/l) concentration of NaSCN required to reduce the starting OD in the ELISA by 50% (IC50). Median and individual responses are shown. Regimens and time-points as in **b** and **c**.

Serum IgG antibody responses against AMA1 were monitored over time in all groups by total IgG enzyme-linked immunosorbent assay (ELISA) against recombinant AMA1 antigen. IgG ELISA antibody units were converted to antigen-specific µg/ml serum antibody concentrations by calibration-free concentration analysis (see Methods and **Supplementary Figure S5**). Median responses in each group are shown over time against FVO AMA1 (**Figure 3a**) as well as individual responses at the peak time-points for both FVO and 3D7 AMA1 (**Figure 3b**,**c**). All 31 volunteers in the study were primed with the same dose of ChAd63 AMA1 on d0 and showed a positive ELISA response at d56 (median: 1.6, range: 0.5–4.0 µg/ml anti-FVO AMA1 IgG) (**Figure 3a**). Following boosting on d56, significantly increased responses were observed across all three vaccinated groups by d84 when the data from the three groups were combined (*P* = 0.0002, *n* = 18, Wilcoxon matched pairs signed-rank test). MVA AMA1 tended to show the lowest responses post-boost on d84 (median of 21.3 µg/ml anti-FVO AMA1 IgG) but this did not reach significance (*P* = 0.29, Kruskal–Wallis test), while the AMA1-C1/Alhydrogel vaccines boosted comparably irrespective of whether CPG 7909 was included in the formulation (medians of 36.4 and 46.3 µg/ml anti-FVO AMA1 IgG, respectively) (**Figure 3a**,**b**). Following boosting on d112, significantly increased responses were again observed across all three vaccinated groups by d140 when the data from the three groups were combined (*P* = 0.0003, *n* = 18, Wilcoxon matched pairs signed-rank test). The highest responses were observed in the AMP+ group with a median response of 87.2 µg/ml anti-FVO AMA1 IgG (four times higher than observed after the first MVA AMA1 boost at week 8). Responses in the A_P+ and A_M groups showed medians of 41.7 and 20.2 µg/ml anti-FVO AMA1 IgG, respectively. Similar to the observations with T cells, there was no difference in antibody immunogenicity following either a MVA AMA1 or AMA1-C1/Alyhdrogel + CPG 7909 vaccine boost at 16 weeks (Groups 5 and 4) in comparison to 8 weeks (Groups 1 and 2, respectively) (**Figure 3a**,**b**).

Responses against the 3D7 allele of AMA1 showed overall similar results at the peak time-points (**Figure 3c**), but anti-3D7 AMA1 serum IgG concentrations tended to be slightly higher for most regimens: medians of 38.4 µg/ml (AM); 67.4 µg/ml (AMP+); 41.4 µg/ml (AP+); 64.0 (AP−); 62.9 µg/ml (A_P+); and 30.9 µg/ml (A_M); also evidenced by an analysis of concordance between the responses against both alleles at the peak of the response (**Figure 3d**). By the final time-point 24 weeks postfinal immunization (d224 or d280), there was no significant difference between any of the regimens, irrespective of number of immunizations, prime-boost interval or adjuvant formulation (*P* = 0.07, Kruskal–Wallis test) (**Figure 3a**).

As part of the same analysis, we also tested nine serum samples that were remaining and available to us from a previous phase 1a vaccine trial of the AMA1-C1/Alhydrogel + CPG 7909 vaccine undertaken in healthy US adults.^37^ These volunteers received two protein-in-adjuvant vaccine immunizations, at the same doses as used here and given 8 weeks apart, thus providing some comparison to the mixed-modality regimens. The responses induced by this protein-only regimen (PP+), as measured in this assay, were median: 131, range: 40–191 µg/ml (anti-3D7 AMA1) and median: 82, range: 25–151 µg/ml (anti-FVO AMA1), and thus higher on average than the other regimens tested here.

### Qualitative assessment of the anti-3D7 AMA1 antibody response

We further assessed qualitative aspects of the anti-3D7 AMA1 serum antibody response following immunization with these various mixed-modality regimens. Initially, we assessed the avidity of the anti-AMA1 IgG antibodies using a NaSCN-displacement ELISA, whereby this is expressed as the concentration of the NaSCN chaotropic agent required to reduce the starting OD in the ELISA by 50%. Results across all of the regimens were largely similar at the peak of the antibody responses (4 weeks postimmunization), irrespective of adjuvant or prime-boost interval (**Figure 3e**). Median responses ranged from 1.3 to 1.7 mol/l, and the results for the ChAd63-MVA AMA1 (AM) regimen here were in very close agreement with data from previous phase 1/2a trials of the same vaccines (Biswas *et al.*, *PLoS One* (in press)).

**Figure 4:**
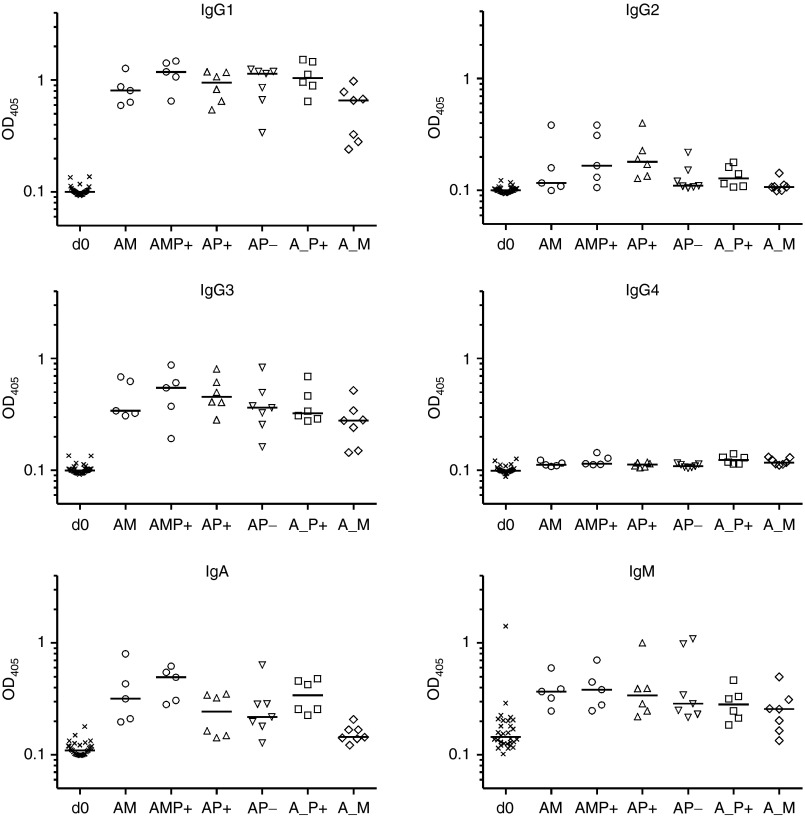
Antibody isotype profiles of mixed-modality AMA1 immunization regimens. Isotype profiles of serum antibody responses were assessed by 3D7 AMA1 enzyle-linked immunosorbent assay (ELISA). Responses are shown at baseline (d0) for all volunteers and then four weeks after all booster vaccinations: day 84 following AM, AP+, and AP− immunization, and day 140 following AMP+, A_P+, and A_M immunization. In all panels, individual and median responses are shown.

In terms of the serum antibody isotype profiles, again the results were largely comparable across all of the tested regimens (**Figure 4**). Anti-3D7 AMA1 IgG responses at the peak of the responses were comprised of a mixed IgG1 and IgG3 response, with low levels of IgG2 in a subset of volunteers and no detectable IgG4. Serum IgA and IgM against AMA1 was also detected for all regimens, although responses were noticeably weaker for the A_M regimen—consistent with this group showing the weakest serum IgG antibody concentrations overall (**Figure 3b**,**c**). Again, results for the AM regimen here were very consistent with those from previous clinical trials of the same vaccines (Biswas *et al.*, *PLoS One* (in press)).

### B cell responses assessed by ELISPOT

**Figure 5:**
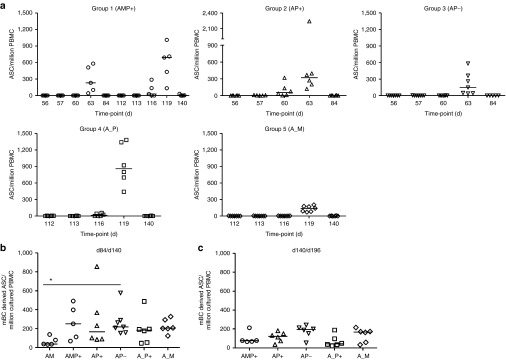
B cell responses of mixed-modality AMA1 immunization regimens. (**a**) AMA1-specific antibody-secreting cell (ASC) responses were assessed in each group by *ex vivo* ELISPOT using 3D7 + FVO AMA1 protein and fresh peripheral blood mononuclear cell (PBMC) from selected time-points post-booster vaccinations (including day of vaccination, and then 1, 4, 7, and 28 days thereafter). Individual and median responses are shown for each group, (note Group 1 received a booster immunization on both d56 and d112). Responses are reported as AMA1-specific ASC / million PBMC used in the assay. Intergroup comparisons, and AMA1-specific ASC reported as % total IgG ASC are shown in **Supplementary Figure S6**. (**b**) AMA1-specific memory B cell (mBC) responses were assessed in each group by ELISPOT assay using 3D7 + FVO AMA1 protein. Frozen PBMC were thawed and underwent a 6-day polyclonal restimulation during which ASC are derived from mBC, before testing in the assay. Responses are shown over time in **Supplementary Figure S7**. Here individual and median responses are reported four weeks after all booster vaccinations: day 84 following AM, AP+, and AP− immunization, and day 140 following AMP+, A_P+, and A_M immunization. (**c**) As for **b**, except the late time-point 12 weeks postfinal immunization is reported (d140/d196). *P < 0.05, Kruskal–Wallis test with Dunn's correction for multiple comparisons.

B cell responses were also monitored over time in all groups. Initially, AMA1-specific antibody-secreting cell (ASC) responses were assessed at key time-points around the time of immunization by *ex vivo* ELISPOT (including day of vaccination, and then 1, 4, 7, and 28 days thereafter) (**Figure 5a**). In all volunteers, AMA1-specific ASC responses peaked 7 days postvaccination, but some lower-level responses were also detected 4 days postvaccination in volunteers receiving AMA1-C1 protein vaccine with CPG 7909. Group 4 (AP+) showed significantly higher responses than Group 3 (AP−) and Group 5 (A_M) (**Supplementary Figure S6a**). These responses also varied as a % of total IgG-secreting ASC, with the lowest median observed in Group 3 (AP−) at 10.7%, and the highest following the AMP+ immunization regimen in Group 1 at 68.0% (**Supplementary Figure S6b**).

mBC responses were also monitored over-time using an established cultured ELISPOT protocol, whereby mBC within PBMC undergo a 6-day polyclonal stimulation to form ASC which are then measured using the same assay protocol as for the *ex vivo* assay. Responses for all volunteers were monitored over-time and are reported as number of mBC-derived AMA1-specific ASC per million cultured PBMC (**Supplementary Figure S7a**), and as a % of total IgG-secreting ASC (**Supplementary Figure S7b**). On average, responses in all groups reached a peak 7–28 days postvaccination. At this time-point, the AM regimen trended to induce the weakest responses, but only one significant difference was noted after correcting for multiple comparisons (**Figure 5b**). At the late time-point (d140/196 = 12 weeks after the final immunization), responses were maintained with no significant differences between any of the five regimens (**Figure 5c**). Over the entire time-course of the study, there were also no notable effects on mBC responses against a bystander antigen—responses against diphtheria toxoid remained stable over time in all groups (**Supplementary Figure S7c**).

### Antibody functional activity: assessment of *in vitro* GIA

**Figure 6:**
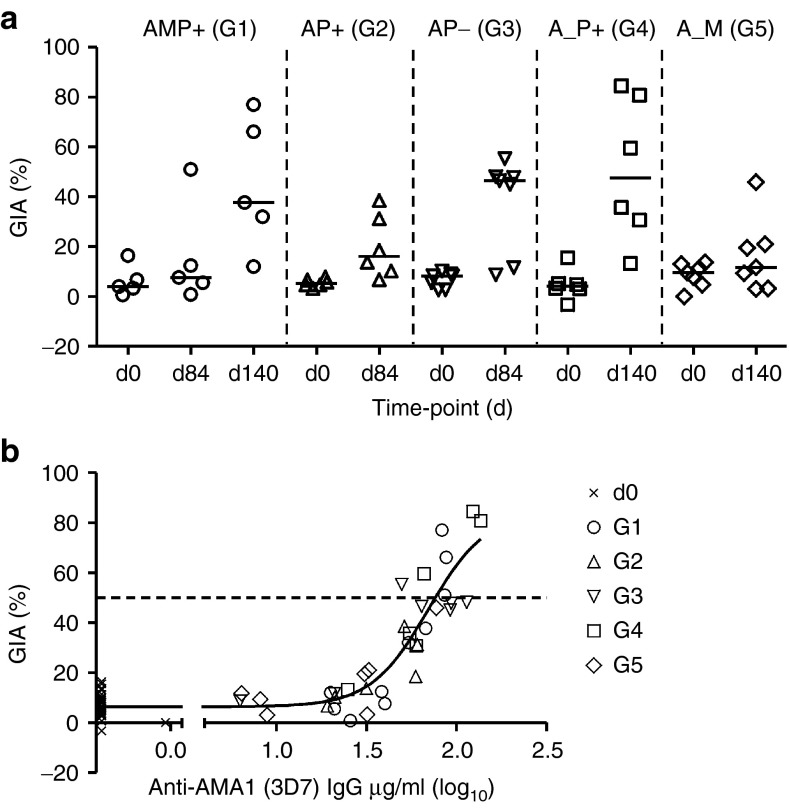
Assessment of functional growth inhibitory activity (GIA) induced by mixed-modality AMA1 immunization regimens. (**a**) *In vitro* GIA of purified IgG was assessed at 10 mg/ml against 3D7 clone *P. falciparum* parasites. Individual data and medians are shown for each group at d0 (baseline), and then 4 weeks following all booster vaccinations (d84 or d140). Responses >20% are typically regarded as positive. (**b**) Relationship between GIA and anti-3D7 AMA1 serum IgG concentrations measured by enzyme-linked immunosorbent assay (ELISA). Nonlinear regression curve is also shown (*n =* 67). The EC_50_ (level of anti-3D7 AMA1 response in this ELISA assay that gives 50% GIA, indicated by the dotted line) was 69.7 µg/ml, (95% CI: 50.2–97.0).

Finally, we assessed the functionality of the anti-AMA1 IgG using the *in vitro* assay of GIA against 3D7 clone *P. falciparum* parasites. IgG was purified from serum at the peak of the antibody responses (4 weeks postimmunization). The highest levels of GIA were observed in the groups receiving the AMP+, AP−, or A_P+ regimens (**Figure 6a**). Overall, there was a strong sigmoidal relationship between % GIA and the anti-3D7 AMA1 serum IgG concentration across all groups, suggesting all five regimens induced a similar quality of functional anti-AMA1 antibody response (**Figure 6b**).

## Discussion

In the absence of safe, effective, scalable, or deployable approaches to whole organism vaccination, subunit vaccines have formed the mainstay approach for the development of novel vaccines against difficult disease targets. Such strategies necessitate the identification of optimal target antigen(s) or immunogens, however, in parallel, it has also been essential to develop effective delivery platforms capable of inducing appropriate and often strong immune responses in humans. To-date, leading approaches have included the use of replication-deficient recombinant viruses (in particular adenoviral and poxviral vectors)—suited for the induction of cellular immunity and moderate levels of antibodies,^18,19,20,38^ as well recombinant protein-in-adjuvant formulations—suited for the induction of strong antibody responses. The latter endeavor requires access to a safe and immunogenic human-compatible adjuvant,^39^ which can be problematic for vaccine developers working on difficult or neglected disease targets.^16^ Moreover, in the field of HIV-1 vaccines, a canarypox (ALVAC) viral prime–protein boost regimen was reported to show low-level efficacy in the RV144 trial,^40^ and future endeavors will likely explore inclusion of adenoviral vaccines as improved priming vectors.^18,41^ Here we report on a phase 1a clinical trial of a “mixed-modality” approach combining adenoviral, poxviral, and protein subunit vaccines targeting the blood-stage malaria antigen *P. falciparum* AMA1.

All volunteers were primed with the ChAd63 AMA1 vector, and then subsequently boosted with MVA AMA1 or AMA1-C1 protein vaccine in adjuvant using either an 8 or 16 week prime-boost interval. Similar to previous reports of these two viral vectors,^22,24^ the ChAd63 and MVA AMA1 vaccines showed a favorable safety profile irrespective of prime-boost time interval. A boost with AMA1-C1 protein in Alhydrogel also showed a very favorable safety profile when given 8 weeks after ChAd63 AMA1. Inclusion of the CPG 7909 with the protein-in-Alhydrogel vaccine led to a small increase in the number of moderate systemic AEs, more similar to ChAd63 AMA1, and this profile was consistent irrespective of prime-boost interval, or whether given 8 weeks after ChAd63 or MVA.

Following ChAd63 priming, T cell responses (detected by *ex vivo* IFN-γ ELISPOT) were measurable in all volunteers and contracted by the time of boosting at 8 or 16 weeks. In agreement with data from mice^25^ and rhesus macaques,^26^ the highest T cell responses were observed 7 days after the MVA AMA1 boost following an 8 week prime-boost interval (AM regimen), while the AMA1-C1 protein vaccine also boosted but to lower levels on average, irrespective of whether CPG 7909 was included in the Alhydrogel adjuvant formulation. Notably, boosting at 16 weeks with either protein or MVA showed a more modest level of T cell boosting, suggesting an 8 week prime-boost interval is preferable. Nevertheless, irrespective of regimen, all responses contracted and there was no significant difference between the five regimens by 24 weeks postfinal immunization. Basic phenotyping of the T cell responses 4 weeks post-boost showed a mixed CD4^+^/CD8^+^ T cell response with no clear differences between regimens, suggesting all delivery modalities (including protein in the relatively weak adjuvant Alhydrogel) could boost the T cell responses primed by ChAd63. It is possible further analyses could reveal more subtle differences in T cell phenotypes induced by MVA as opposed to protein-in-adjuvant vaccine boosting. Interestingly, a previous study using the same protein vaccine and ELISPOT assay reported an average of 198 AMA1 (3D7)-specific SFU/million PBMC 14 days after a single immunization with AMA1-C1 Alhydrogel + CPG 7909, and 282 SFU/million PBMC 14 days after a second immunization using an 8-week prime-boost interval (*i.e.*, the PP+ regimen), and these T cells were predominantly CD4^+^.^35^ These data thus suggest that boosting with the protein vaccine after priming with ChAd63 (as opposed to priming with protein) leads to stronger cellular immune responses, including CD8^+^ T cells.

In the case of serum antibody responses, boosting with the protein-in-adjuvant vaccine tended to lead to the highest anti-AMA1 IgG concentrations, again in agreement with preclinical data^25,26,27^ and suggesting the protein-in-adjuvant boost is more effective than MVA in humans. There was no discernable benefit of boosting at 16 weeks as opposed to 8 weeks, nor did an intervening MVA administration prior to the protein vaccination (the AMP+ regimen) significantly improve overall responses in comparison to the AP−, AP+, or A_P+ regimens. These results are highly comparable to previous data with very similar AMA1 vaccines tested in rhesus macaques, whereby the highest titers of antibody were observed following AMP+ or A_P+ immunization when using Alhydrogel or the more potent CoVaccineHT adjuvant.^26^ Encouragingly, the AP− regimen tested here performed comparably to the AP+ regimen, with the AMA1-C1 Alhydrogel boost inducing on average 46 and 64 µg/ml anti-FVO and -3D7 AMA1 IgG respectively, (about twofold higher than the concentrations achieved by the AM regimen). These data confirm previous observations in mice that suggested adenoviral vaccine priming led to comparable boosting of antibody responses by protein-in-adjuvant vaccines, irrespective of the adjuvant or its relative potency when tested in a protein-only regimen.^27^ It will also remain of interest in future studies to explore the merits of multiple protein-in-Alhydrogel booster immunizations to see whether peak antibody concentrations can be further improved by such a strategy.

Previous data in mice using MSP1 vaccines had suggested inclusion of both viral vectors in the immunization regimen may improve IgG avidity.^25^ Here with AMA1 vaccines in humans, avidity was largely comparable across all regimens tested, irrespective of whether they included a boost with MVA. Further studies will be required to assess for more subtle differences in the quality of the IgG response, in terms of the possible differences at the level of the antibody repertoire, epitope fine specificity and degree of somatic hypermutation related to the induction of germinal centers and CD4^+^ T follicular helper cell responses. Nevertheless, there remained a strong relationship between serum anti-AMA1 IgG concentration and functional GIA *in vitro* across all regimens, suggesting no major differences in the quality of vaccine-induced IgG. The GIA EC_50_ defined here against 3D7 clone parasites (69.7 µg/ml anti-3D7 AMA1 IgG) was also in agreement with previous reports for the AMA1-C1 vaccine tested in malaria-naive adults.^7,36,42,43^ Similar to avidity, the antibody isotype profiles were largely comparable across all regimens, with IgG1 and IgG3 the main isotypes induced against AMA1. In contrast, the same protein in Alhydrogel administered to US adults induced predominantly IgG1 alone.^44^ Induction of both Th1-type human IgG isotypes (IgG1 and IgG3) is consistent with adenoviral priming, as observed in mice where responses are skewed from predominantly IgG1 to both IgG1 and IgG2a when using viral vectors in conjunction with adjuvants such as Alhydrogel.^27^

Previous studies with the AMA1-C1 vaccine reported significant improvements in B cell memory following addition of CPG 7909 to the Alhydrogel adjuvant in US adults,^45^ but surprisingly not in Malian adults.^46^ In our study, all regimens tested produced largely comparable AMA1-specific mBC responses, both at the peak of the response and 12 weeks postfinal immunization. These responses were largely comparable to those previously reported for the AM regimen and also the PP+ regimen in UK and US adults respectively,^23,45^ again confirming that the protein vaccine in Alhydrogel (AP− regimen) is capable of boosting such responses once primed by ChAd63. Similar to other studies,^23,47^ AMA1-specific ASC responses were also consistently detectable 7 days post-booster immunization irrespective of whether MVA or protein was used. Notably, inclusion of CPG 7909 tended to lead to earlier detection of peripheral AMA1-specific ASC responses (on day 4 post-boost) albeit at much lower levels as compared to day 7.

This study aimed to assess whether any of these “mixed-modality” vaccine delivery regimens were largely superior in terms of antibody induction. A comparison (using the same ELISA assay) to historical sera that were available from US adults immunized with the PP+ regimen,^37^ suggests that protein vaccine formulated in a potent adjuvant remains the most immunogenic delivery platform. Indeed, antibody responses reported for this regimen here were median 131 µg/ml (anti-3D7 AMA1) and 82 µg/ml (anti-FVO AMA1), and largely comparable to other reports for the same vaccine tested with similar regimens elsewhere.^35,36^ Nevertheless, the same AMA1-C1 vaccine delivered in Alhydrogel alone in US adults induced on average only 5–20 µg/ml anti-AMA1 IgG^36,48^ and 16% GIA against 3D7 parasites,^36^ and thus it remains encouraging that the AP− regimen reported here improved on those reported data. Other AMA1 protein vaccines formulated with strong adjuvants (such as Montanide ISA 720, AS01B and AS02A), have also induced high-serum IgG concentrations and consequently high level of *in vitro* GIA,^42,49^ and one candidate has shown evidence of strain-specific efficacy in a phase 2b field trial in Malian children^50^; while another novel candidate in preclinical development has shown an improved quality of vaccine-induced anti-AMA1 IgG response.^51^ It thus remains important in future clinical studies to assess whether anti-AMA1 IgG and/or *in vitro* GIA associate with protective outcome,^35,52,53^ in order to guide the onward clinical development of blood-stage malaria vaccine candidates.

Access to potent human-compatible adjuvants is not always easy for vaccine developers working on neglected diseases, and similarly, experimental unlicensed adjuvants, although potent, may show unacceptable side effects or reactogenicity during clinical development. CPG 7909 has also shown reduced potency in African adults (as compared to US adults) when formulated with AMA1-C1 in Alhydrogel.^36,54^ Moreover, even though exceptionally high antibody concentrations are likely required to neutralize merozoite invasion of red blood cells (a level not achieved by the AMA1-C1/Alhydrogel + CPG 7909 vaccine in UK adults),^35^ other disease targets may be inherently more susceptible, *e.g.*, anti-capsular antibody concentrations of >0.35 µg/ml are regarded as sufficient for protection against invasive pneumococcal disease.^55^ Consequently, the ability to improve the potency of an aluminium-based adjuvant by priming with an adenoviral vaccine vector encoding the same antigen may find utility against other malaria antigens or disease targets; especially where the use of aluminium-based salts alone has proved insufficiently immunogenic, or where access to more potent adjuvants is not possible or their use is undesirable in the target population.

## Materials and Methods

***Participants.*** The VAC044 study was conducted at the Oxford Vaccine Centre, part of the Centre for Clinical Vaccinology and Tropical Medicine, University of Oxford, UK. Healthy, malaria-naive males and nonpregnant females aged 18–50 years were invited to participate in the study. All volunteers gave written informed consent prior to participation, and the study was conducted according to the principles of the Declaration of Helsinki and in accordance with Good Clinical Practice. There was no selection of volunteers on the basis of pre-existing neutralizing antibodies to the ChAd63 vector prior to enrolment (see **Supplementary Material** for the full list of inclusion and exclusion criteria).

***Ethical and regulatory approval.*** All necessary approvals for VAC044 were granted by Oxfordshire Research Ethics Committee A (Ref:11/H0604/2) and the UK Medicines and Healthcare products Regulatory Agency (Ref: 21584/0280/001-0001). Vaccine use was authorized by the Genetically Modified Organisms Safety Committee of the Oxford University Hospitals NHS Trust (Reference number GM462.10.59). The trial was registered with ClinicalTrials.gov (Ref: NCT01351948). The Local Safety Committee provided safety oversight and Good Clinical Practice compliance was independently monitored by an external organization (Appledown Clinical Research, Great Missenden, UK).

***Vaccines.*** Generation, manufacture, and QC testing of the recombinant ChAd63 and MVA vectors encoding AMA1 has been previously described.^22,26,56^ Briefly, the AMA1 transgene insert contains from N- to C-terminus: the leader sequence from human tissue plasminogen activator followed in-frame by sequence encoding the ectodomain of *P. falciparum* (clone 3D7) AMA1 linked to the ectodomain plus C-terminal transmembrane region of *P. falciparum* (strain FVO) AMA1. In the case of the recombinant protein-in-adjuvant vaccines, details of the manufacture and formulation of both AMA1-C1/Alhydrogel and CPG 7909 in saline, and the mixing procedure used in the clinic, have been described in detail elsewhere.^37,57^ Briefly, the AMA1-C1 vaccine contains an equal mixture of two 533 amino acid recombinant malaria proteins based on the AMA1 sequences of the FVO strain and 3D7 clone of *P. falciparum*. The recombinant proteins consist of the correctly folded ectodomain portions of the antigens, with the addition of a six-histidine C-terminal tag to enable protein purification, and are expressed separately in *Pichia pastoris*. CPG 7909 was provided by Pfizer (New York, NY).

***Study design.*** This was a phase 1a open-label, nonrandomized observational and descriptive vaccine trial to assess the safety and immunogenicity of ChAd63 AMA, MVA AMA1, and AMA1-C1/Alhydrogel ± CPG 7909 administered in various regimens. Allocation to study groups (aiming for *n* = 6–7 per group) (**Figure 1**) occurred at screening based on volunteer preference as previously described.^21^ All vaccinations were administered intramuscularly (IM) into the deltoid, with sequential vaccines administered into the deltoid of alternating arms. Details of dosing, clinical follow-up and safety monitoring are given in Supplementary Methods. Throughout the paper, study day refers to the nominal time-point for a group and not the actual day of sampling.

***PBMC and serum preparation.*** Blood samples were collected into lithium heparin-treated vacutainer blood collection systems (Becton Dickinson, Oxford, UK). PBMC were isolated and used within 6 hours in fresh assays as previously described.^21^ Excess cells were frozen in fetal calf serum containing 10% dimethyl sulfoxide (DMSO) and stored in liquid nitrogen. For serum preparation, untreated blood samples were stored at 4 °C and then the clotted blood was centrifuged for 5 minutes (1,000 *×g*). Serum was stored at −80 °C. Historical serum samples from nine healthy US volunteers previously immunized with AMA1-C1/Alhydrogel + CPG 7909 were the only ones available and were provided to us from a previous trial.^37^ These volunteers were immunized twice, 8 weeks apart, with the same doses of vaccine as used in this study, and this regimen is referred to as “PP+”.

***Ex vivo interferon-γ (IFN-γ) ELISPOT.*** The kinetics and magnitude of the T cell response to AMA1 were assessed over time by *ex vivo* IFN-γ ELISPOT following an 18–20 hour restimulation of PBMC with overlapping peptides spanning the entire AMA1 sequence present in the vaccines. Peptides were purchased from NEO Peptide (Cambridge, MA) and the sequences have been previously described.^22,24^ 20mer peptides overlapping by 10 amino acids (aa) were generated for the whole of the bi-allelic AMA1 vaccine insert present in the ChAd63 and MVA vaccines which also matched the two AMA1 alleles in the AMA1-C1 protein vaccine.^26,56^ Peptides were reconstituted in 100% DMSO at 50–200 mg/ml and combined into various pools for ELISPOT and flow cytometry assays as previously described.^24^ In brief, for the ELISPOT, peptides were divided into pools containing up to 10 peptides per pool according to whether they were 3D7-specific, FVO-specific, common peptides, or FVO C-terminus peptides. Fresh PBMC were used in all ELISPOT assays using a previously described protocol.^21^ Spots were counted using an ELISPOT counter (Autoimmun Diagnostika (AID), Strasberg, Germany). Results are expressed as IFN-γ spot-forming units per million PBMC. Background responses in unstimulated control wells were almost always less than 20 spots per 250,000 cells, and were subtracted from those measured in peptide-stimulated wells. Responses are reported as the 3D7 allele-specific AMA1 response (summed response for 3D7-specific peptides + common peptides + C-terminal tail peptides).

***Multiparameter flow cytometry.*** Cytokine secretion by PBMC was assayed by intracellular cytokine staining followed by flow cytometry using a previously reported protocol^22^ with some minor changes. Briefly, frozen PBMC were restimulated for 18 hours in the presence of anti-human CD49d and CD28 (BD Biosciences, Oxford, UK) and CD107a. Restimulation for the final 16 hours was carried out in the presence of Brefeldin A (Sigma, Gillingham, UK) and Monensin (Golgi Stop, BD Biosciences). Each sample was restimulated with either: 1 µg/ml Staphylococcal enterotoxin B (positive control samples); a single pool of 3D7 AMA1-specific peptides (*n* = 56) at final concentration 2 µg/ml each peptide (0.11% total DMSO concentration); and 0.11% DMSO final concentration (unstimulated peptide control sample). Cells were stained the next day using a Live/Dead marker, as well as for CD4, CD14, CD19, CD8α, CD3, IFN-γ, TNF-α, and IL-2. The staining antibodies differed from those previously reported only for CD3 (Alexa 700; clone: UCHTI) and CD19 (eFluor450; clone: HIB19) (eBioscience, Hatfield, UK). Samples were analyzed using a LSRII Flow Cytometer (BD Biosciences) and FlowJo v9.7.5 (Tree Star; FlowJo, Ashland, OR). Dead cells, monocytes (CD14+), and B cells (CD19+) were excluded from the analysis (**Supplementary Figure S4**). Background responses in unstimulated no peptide control cells were subtracted from the antigen-specific peptide responses.

***Total IgG ELISA.*** Recombinant 3D7 AMA1 protein was produced in HEK293E cells. Briefly, suspension HEK293E cells were transiently transfected with an expression plasmid encoding: the ectodomain of 3D7 AMA1 (amino acids 25–546, with sites of potential N-linked glycosylation removed as previously described),^26^ with the human tissue plasminogen activator signal peptide fused to the N-terminus,^17^ and the AviTag biotin acceptor peptide (amino acids GLNDIFEAQKIEWHE) followed by a hexa-histidine (His6) tag fused to the C-terminus. When cell viability fell below 95%, culture supernatants were harvested and the recombinant protein was purified in a single step by immobilized metal ion (Ni++) chromatography and quantified by nanodrop. The recombinant FVO AMA1 protein used for ELISAs was a gift from Dr Mike Blackman (NIMR, London, UK).^56^ Total IgG ELISAs were performed with these proteins using standardized methodology, as previously described.^22^ The limit of detection in the ELISA assays for both alleles of AMA1 was 10 AU, and we assigned the AU value of 1.0 for any test samples with less than 10 AU.

***Calibration-free concentration analysis (CFCA).*** AMA1 IgG antibody OD-based ELISA units were converted to antigen-specific µg/ml as follows. CFCA was performed with a method similar to that previously described,^58^ using a Biacore T200 instrument, a Biotin CAP chip, and T200 control and evaluation software (all from GE Lifesciences, Amersham, UK). Recombinant 3D7 and FVO AMA1 proteins were produced by generating plasmids encoding each allele's full-length AMA1 ectodomain sequence (identical to those included in both the AMA1-C1 protein^57^ and viral vector vaccines, and reported elsewhere).^26,56^ Sequences were codon-optimized for human expression and possessed an N-terminal human tissue plasminogen activator signal peptide and C-terminal AviTag (as above) and StrepII tag. Protein was produced as described above by transient transfection of HEK293E cells; both antigens were enzymatically monobiotinylated by cotransfection of the cultures with a plasmid encoding BirA,^59^ then dialysed extensively against PBS prior to CFCA. The CFCA was performed using day 84 serum samples from three individuals with a range of ELISA-measured AMA1-specific IgG antibody responses. Two replicate dilutions of each individual's sera (1:250 in running buffer)^58^ were prepared and assayed independently on different days. Mass-transport limited binding conditions were obtained by capturing a minimum of 800 response units (RU) of AMA1 antigen on the active flow cell. The chip was regenerated with the manufacturer's supplied regeneration and CAP reagents and fresh antigen prior to each application of antibody; variation in the level of antigen capture between cycles was typically <2%. Antigen-specific antibody binding was measured by double reference subtraction, firstly of binding to a flow cell coated only with the biotin capture reagent, and secondly of the binding of the same individual's day 0 serum sample from that of the day 84 sample (**Supplementary Figure S5a–c**). Initial rates of antigen-specific binding at 5 µl/minute and 100 µl/minute were measured and compared to permit measurement of concentration and the level of mass-transport limitation. The binding model used a molecular weight of 150 kDa for IgG and a diffusion coefficient of IgG under the test conditions (37 °C, running buffer) of 5.5 × 10^−11^ m^2^/s.^58^ Initial binding rates were in the range 0.23–1.24 RU/s at 5 µl/minute flow, and calculated quality control ratios exceeded the manufacturer's recommended value of >0.13 (reflecting adequate mass transport limitation for concentration estimation). The CFCA-measured antigen-specific antibody concentrations for each individual against 3D7 and FVO sequence AMA1 were combined with the known total IgG ELISA AU measurements for the same samples to derive an AU-to-µg/ml conversion factor. For each AMA1 allele, the mean of the conversion factors measured for the three subjects was calculated and applied to express other subjects' ELISA results in terms of µg/ml units. Conversion factors calculated using CFCA results (**Supplementary Figure S5d**) were 0.014 µg/ml per anti-3D7 AMA1 IgG antibody unit, and 0.039 µg/ml per anti-FVO AMA1 IgG antibody unit.

It should be noted that a proportion of the binding detected by the CFCA assay is likely to be due to AMA1-specific IgA and IgM. Given that the levels of AMA1-specific IgA and IgM are on the margins of detectability by ELISA whereas AMA1-specific IgG is detectable in serum diluted many 1,000-fold, the substantial majority of the CFCA binding detected is likely to be attributable to AMA1-specific IgG. We therefore used the CFCA measurement of antigen-specific antibody concentration as a conversion factor for the IgG ELISA. To add support to this approach, we also tested eight serum samples from a previous phase 1a trial (VAC036) of ChAd63-MVA AMA1. In the previous trial, the IgG antibody responses in these eight sera were converted to AMA1-specific µg/ml IgG concentrations using a conversion factor assigned to an IgG reference standard prepared by affinity-purification on AMA1 antigen (as reported in our previous study).^22^ Here, we now remeasured these eight samples by ELISA and converted them to AMA1-specific IgG antibody concentrations using the conversion factor from the CFCA. There was a strong concordance between the results (**Supplementary Figure S5e,f**).

***IgG antibody avidity.*** IgG antibody avidity was assessed by sodium thiocyanate (NaSCN)-displacement ELISA as described in detail elsewhere (Biswas *et al.*, *PLoS One* (in press)). In brief, the assays were performed using 3D7 AMA1 protein exactly as for total IgG except sera were individually diluted to a level calculated to give an OD_405_ = 1.0, plated and then exposed to an ascending concentration of the chaotropic agent NaSCN down the plate (0–7 mol/l NaSCN). Plates were incubated for 15 minutes before washing and development as for total IgG. The concentration of NaSCN required to reduce the OD_405_ to 50% of that without NaSCN was used as a measure of avidity.

***Antibody isotype ELISA.*** Antibody isotype ELISAs were performed as described in detail elsewhere (Biswas *et al.*, *PLoS One* (in press)). In brief, the assays were performed using 3D7 AMA1 protein exactly as for total IgG except sera were individually diluted 1:100 and added to duplicate wells of six 96-well plates (one for each isotype analysis). After incubating for 2 hours, the plates were washed and the secondary antibodies added for human IgG1, IgG2, IgG3, IgG4, IgA, and IgM. After incubation and washing, plates were developed as per the total IgG ELISA. Blank wells, internal development controls, and a series of positive monoclonal antibody controls were included on each plate.

***B cell ELISPOT assays.*** B cell ELISPOT assays were performed as described in detail elsewhere.^23^ In brief, to measure mBC responses, frozen PBMC were thawed before culturing with a polyclonal B cell stimulation mix containing *Staphylococcus aureus* Cowan strain Pansorbin cell “SAC” (Calbiochem; Merck Millipore, Watford, UK), the human TLR agonist CpG ODN-2006 (Invivogen, Toulouse, France) and pokeweed mitogen “PWM” (Sigma) for 6 days, allowing mBC to differentiate into ASC. On day 5 of the experiment, ELISPOT plates were coated with recombinant AMA1 protein (a 50:50 mixture of the 3D7 and FVO alleles) to measure the antigen-specific response and polyvalent goat-anti human IgG (Caltag) to measure the total IgG response. A separate plate was coated with a nonmalaria vaccine antigen (diphtheria toxoid (DT)), and PBS-coated wells were used as a negative control. On day 6, cultured cells were transferred to the ELISPOT plate and incubated for 18–20 hours before developing with an anti-human IgG (γ-chain) antibody conjugated to alkaline phosphatase (Calbiochem) followed by a substrate buffer. Plates were counted using an AID ELISPOT plate reader. *Ex vivo* ASC ELISPOT assays were performed exactly as above but using fresh PBMC directly prepared and added to the ELISPOT plate with no preceding 6-day culture.

**In vitro *assay of GIA.*** The ability of induced anti-AMA1 antibodies to inhibit growth of *P. falciparum* 3D7 clone parasites *in vitro* were assessed by a standardized GIA assay using purified IgG as previously described.^7^ Briefly, each test IgG (10 mg/ml in a final test well) was incubated with synchronized *P. falciparum* parasites for a single growth cycle and relative parasitemia levels were quantified by biochemical determination of parasite lactate dehydrogenase.

***Statistical analysis.*** Data were analyzed using GraphPad Prism version 5.04 for Windows (GraphPad Software, San Diego, CA). Median responses for each group are described. Significance testing of differences between groups used the Kruskal–Wallis test with Dunn's correction for multiple comparisons, or the Wilcoxon matched-pairs signed rank test was used when data were paired (*e.g.*, between time-points). To analyze concordance, linear regression was performed without constraints but with automatic removal of outliers. A value of *P* < 0.05 was considered significant.

**SUPPLEMENTARY MATERIAL** **Figure S1.** Local and systemic AEs deemed definitely, probably or possibly related to ChAd63 AMA1. **Figure S2.** Local and systemic AEs deemed definitely, probably or possibly related to MVA AMA1. **Figure S3.** Local and systemic AEs deemed definitely, probably or possibly related to AMA1-C1/Alhydrogel ± CPG 7909. **Figure S4.** Gating strategy for the analysis of antigen-specific T cell responses. **Figure S5.** Calibration-free concentration analysis (CFCA). **Figure S6.** *Ex-vivo* ASC responses following mixed-modality AMA1 immunization regimens. **Figure S7.** mBC responses over time.

## Supplementary Material

Supplementary Figure S1 Local and systemic AEs deemed definitely, probably or possibly related to ChAd63 AMA1.

Supplementary Figure S2 Local and systemic AEs deemed definitely, probably or possibly related to MVA AMA1.

Supplementary Figure S3 Local and systemic AEs deemed definitely, probably or possibly related to AMA1-C1/Alhydrogel ± CPG 7909.

Supplementary Figure S4 Gating strategy for the analysis of antigen-specific T cell responses.

Supplementary Figure S5 Calibration-free concentration analysis (CFCA).

Supplementary Figure S6 *Ex-vivo* ASC responses following mixed-modality AMA1 immunization regimens.

Supplementary Figure S7 mBC responses over time.
