# Supplementary figures and images for: Combining Viral Vectored and Protein-in-adjuvant Vaccines Against the Blood-stage Malaria Antigen AMA1: Report on a Phase 1a Clinical Trial
Source: Mol Ther. 2014 Sep 30;22(12):2142–54. doi: 10.1038/mt.2014.157 (PMC4250079; doi:10.1038/mt.2014.157)

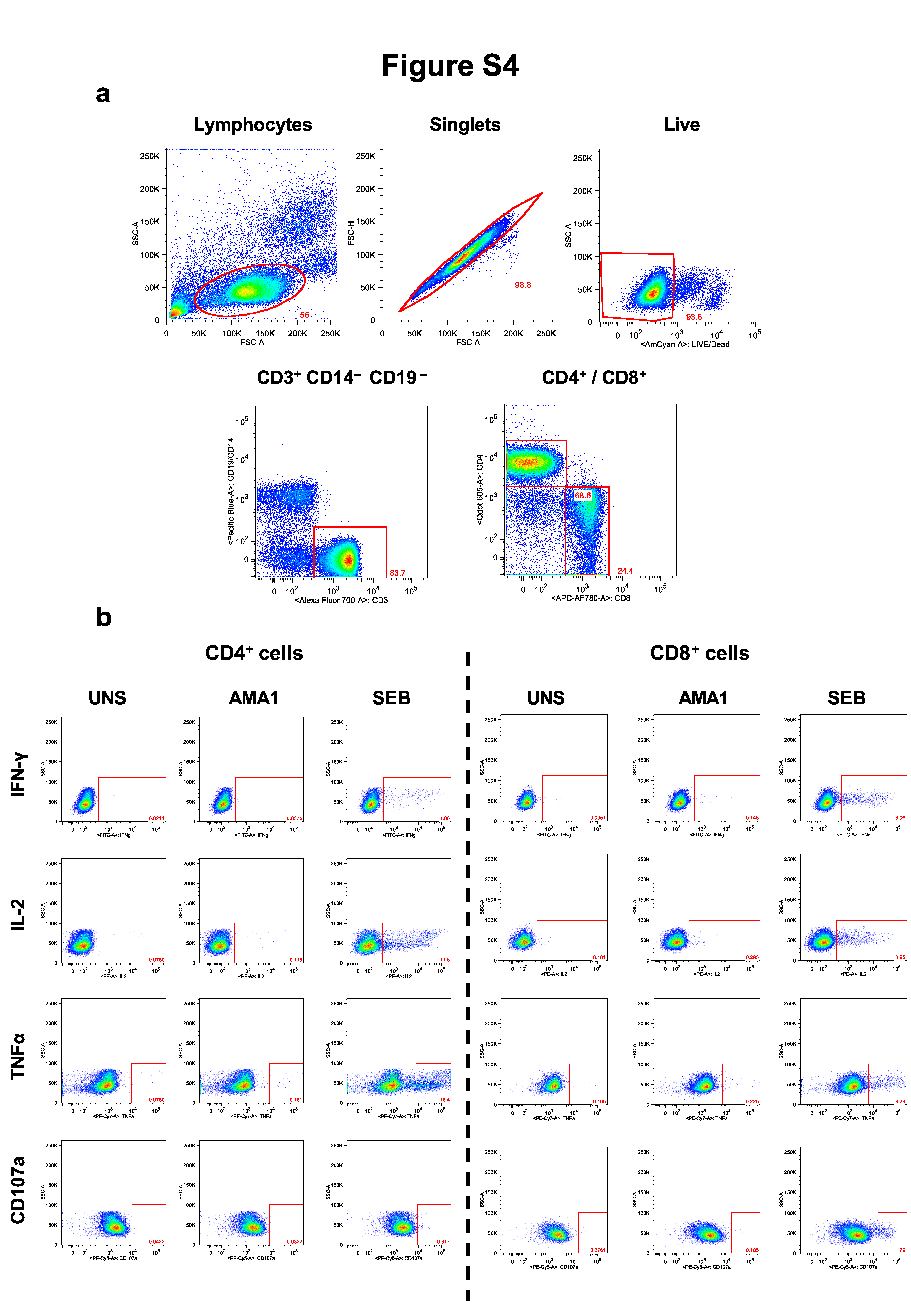

Supplement: Supplementary Figure S4 — Gating strategy for the analysis of antigen-specific T cell responses. [file mt2014157x4.tiff]
